# Assessing Humoral Immuno-Inflammatory Pathways Associated with Respiratory Failure in COVID-19 Patients

**DOI:** 10.3390/jcm12124057

**Published:** 2023-06-15

**Authors:** Matteo Regolo, Alessandra Sorce, Mauro Vaccaro, Michele Colaci, Benedetta Stancanelli, Giuseppe Natoli, Massimo Motta, Ivan Isaia, Federica Castelletti, Federica Giangreco, Daniela Fichera, Paola Aparo, Alessandra Lanzafame, Mario Russo, Nicola Santangelo, Paola Noto, Lorenzo Malatino

**Affiliations:** 1 Department of Clinical and Experimental Medicine, University of Catania, 95123 Catania, Italy; matteo.regolo.94@gmail.com (M.R.); michele.colaci@unict.it (M.C.); massimo.motta@unict.it (M.M.); ivanisaia92@gmail.com (I.I.); federica.castelletti1@gmail.com (F.C.); p_aparo@hotmail.it (P.A.); alessandralanzafame.512@gmail.com (A.L.); 2 Academic Unit of Internal Medicine, Cannizzaro Hospital, 95126 Catania, Italy; giuseppenatoli@hotmail.it (G.N.); federicagiangreco90@gmail.com (F.G.); russom32@gmail.com (M.R.); nicola.santangelo@libero.it (N.S.); 3 Department of Health Promotion Sciences, Maternal and Infant Care, Internal Medicine and Medical Specialties, “G. D’Alessandro” (PROMISE), Unit of Nephrology and Hypertension, European Society of Hypertension Excellence Centre, University of Palermo, 90133 Palermo, Italy; avinouros85@gmail.com; 4 Department of Emergency Medicine, San Marco-Polyclinic Academic Hospital, 95121 Catania, Italy; maurovaccaro90@gmail.com (M.V.); daniela.fichera12@gmail.com (D.F.); p.noto@hotmail.it (P.N.); 5 Unit of Internal Medicine, San Marco-Polyclinic Academic Hospital, 95121 Catania, Italy; benedetta.stancanelli@virgilio.it

**Keywords:** NLR, CRP, P/F, COVID-19, neutrophil-to-lymphocyte ratio, neutrophils, lymphocytes, immune system, biomarkers, SARS-CoV-2, inflammation, ICU, lung failure

## Abstract

All severe cases of SARS-CoV-2 infections are characterized by a high risk of disease progression towards ARDS, leading to a bad outcome. Respiratory symptoms in COVID-19 patients often do not correspond to disease’s worsening. In our sample, median age was 74 years (72–75) and 54% were men. The median period of hospitalization was 9 days. Firstly, we observed a significant asynchronous trend of neutrophil-to-lymphocyte ratio (NLR) and C-reactive protein (CRP) in 764 selected among 963 patients, who were consecutively recruited in two hospitals (Cannizzaro, S. Marco) in Catania, Italy. NLR values in deceased patients showed an increase from baseline over time. By contrast, CRP tended to fall from baseline to median day of hospitalization in all three subgroups, but steeply increased at the end of hospitalization only in ICU-admitted patients. Then, we evaluated the relationships between NLR and CRP as continuous variables with PaO_2_/FiO_2_ ratio (P/F). NLR was an independent predictor of mortality (HR: 1.77, *p* < 0.0001), while ICU admission was more significantly associated with CRP (HR: 1.70, *p* < 0.0001). Finally, age, neutrophils, CRP, and lymphocytes are significantly and directly linked to P/F, while the influence of inflammation on P/F, reflected by CRP, was also mediated by neutrophils.

## 1. Introduction

The clinical features of SARS-CoV-2 infection are various, from mild to moderate symptoms, characterized by spontaneous regression to severe cases, and showing conditions leading to ICU admission or death [1,2]. Despite the fact that the virus’ transmission and clinical presentation are well known, scant information is available about the pathophysiology of disease worsening.

The severe respiratory failure caused by COVID-19 is primarily manifested as acute respiratory distress syndrome (ARDS). Its related pathological findings were often documented in most COVID-19 autoptic studies [3]. ARDS nosography has been substantially modified after the COVID-19 pandemic, since several studies have shown that COVID-19-related ARDS is atypical, although often falling within the Berlin definition of ARDS [4,5].

Inflammation plays a key role in the disease worsening, if any, of COVID-19 patients. Several studies have focused on the cytokine storm and the lymphocyte fall observed in these patients [6,7,8]. The relationship between the clinical presentation of severe cases of COVID-19 and the related immuno-inflammatory factors are not, so far, well understood [9].

The early identification of cases with greater clinical severity is mandatory to ensure the best possible care. The detection of laboratory biomarkers or indices representing the unbalanced innate and adaptive immune responses allows a rapid characterization of patients in a hospital setting. Venous and arterial samplings, together with a chest computer tomography (CT) could quickly provide enough information to recognize hyper-inflammatory states and respiratory distress, assess clinical severity, and predict outcome.

Several biochemical parameters were under investigation for an early assessment of disease severity [10,11]. Among them, C-reactive protein (CRP) and neutrophil-to-lymphocyte ratio (NLR) are rapid and widely available inflammatory indices, recently under debate as predictors of a bad outcome in a large variety of pathological conditions characterized by exaggerated systemic inflammation [12,13]. In our previous study we found that both NLR and CRP are reliable prognostic predictors in COVID-19 patients [14].

Neutrophilia, lymphopenia, and high CRP levels are closely linked with the pathophysiology of COVID-19. A cytokine storm involves a massive recruitment of circulating cells, resulting in a continuous activation of neutrophils, incremental consumption, negative counter-regulation of lymphocytes and a strengthened inflammatory response, with an over-production of CRP, which is a protein of acute phase.

In the present study, we aimed to evaluate the time course of immuno-inflammatory pathways’ involvement in the development of respiratory failure. We especially evaluated the dynamic changes of neutrophils, lymphocytes, and CRP during hospitalization. Finally, we assessed the link between CRP, neutrophils, and lymphocytes with P/F ratio to better understand the individual role of mediators leading to respiratory failure.

## 2. Materials and Methods

**Figure 1:**
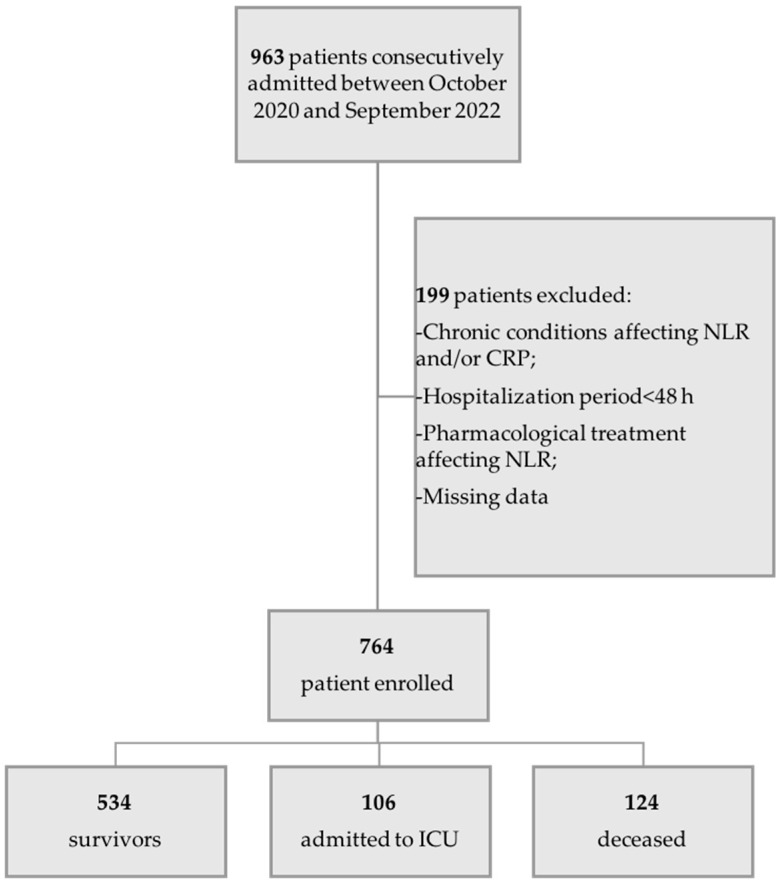
Flow chart of the protocol and subsets of patients divided for outcome. NLR: neutrophil-to-lymphocyte ratio.

In this retrospective, multi-center, observational study, a total of 963 patients were consecutively candidates among patients admitted to the Emergency Unit of San Marco Hospital and the COVID-19 Internal Medicine Unit at Cannizzaro Hospital, in the city of Catania, Italy, between October 2020 and September 2022. Within this cohort, 764 patients were selected based on predefined inclusion criteria (Figure 1):

(1) A reliable diagnosis of SARS-CoV-2 infection obtained by RT-PCR molecular swab testing.
(2) No history of pharmacological treatments responsible for alterations in the leukocyte count and/or CRP upon admission.
(3) No current or past history of conditions responsible for alterations in the leukocyte count and/or CRP.
(4) Availability of at least three blood tests and blood gas analyses during hospitalization, and a hospitalization period not less than 48 h.

Patients with previous or intercurrent bacterial overlap contributing to outcome were excluded, as well as patients who died from specific causes unrelated to SARS-CoV-2 infection (sepsis, ischemic heart disease).

Routine biomarkers were measured with standard techniques using auto-analyzers (Beckman Coulter DxH 800; Danaher Corporation, Miami, FL, USA, Beckman DxC 700 AU; Danaher Corporation, Miami, FL, USA).

Total and differential leukocyte count and CRP levels were measured three times: at baseline, on the median day of hospitalization, and at discharge (defined as death, transfer to the Intensive Care Unit, ICU, or routine discharge).

The severity of respiratory failure was identified using the P/F ratio obtained by blood gas test reports using PaO_2_/FiO_2_ ratio, namely, the ratio between partial arterial pressure of oxygen and the fraction of inspired oxygen. Baseline P/F values were used for statistical analyses. Baseline oxygen flow rates and FiO_2_ values of oxygen supplementation are specified in Table 1.

The neutrophil-to-lymphocyte ratio (NLR) was calculated as follows: NLR = number of neutrophils/number of lymphocytes.

Relevant information was obtained by reviewing the medical records of the participants, including their demographic and anamnestic data, clinical and laboratory characteristics, treatment regimen, and outcome.

Appropriate measures were taken to display information before performing statistical analysis.

The present study exclusively focused on death following SARS-CoV-2 infection, excluding deaths caused by any other factors.

### Statistical Analysis

The statistical analysis was performed using the IBM-SPSS (version 28.0.1.1) and R statistical software packages (version 4.3.0).

Categorical variables were described in terms of absolute frequency and percentage prevalence, while continuous variables were further divided into two groups after performing the Kolmogorov–Smirnov test to evaluate their distribution. Continuous variables were expressed as mean ± standard deviation in the presence of normal distribution and median and interquartile range in the presence of non-Gaussian distribution.

The sample was initially divided into three groups, according to outcome. Differences between outcomes were evaluated using the chi-square test with Fisher’s correction for categorical variables, one-way ANOVA for normally distributed continuous variables, and the Kruskal–Wallis test for non-normally distributed continuous variables.

The null hypothesis was excluded, in all two-tailed tests, for *p* values < 0.05.

Non-parametric tests, such as Spearman’s correlation coefficient and Kendall’s Tau, were used to assess the correlations between the variables of interest, considering their distribution.

Where appropriate we used the z-scores for variables characterized by extreme kurtosis and asymmetry.

Firstly, to evaluate the relationship between NLR and CRP as continuous variables with PaO_2_/FiO_2_ ratio, univariate and multivariate logistic regression models were used and odds ratios (ORs), both corrected and uncorrected, as well as 95% confidence intervals (CIs) were calculated.

All of these relationships were assessed in the whole sample and according to single outcome.

Multiple logistic and Cox regression models were built to verify the associations between biomarkers and outcome.

Repeated ANOVA measures were performed, whenever necessary, to test the differences over time in the mean levels of variables.

Finally, to better understand the existing relationships between inflammatory biomarkers and P/F ratio, we performed a mediation analysis to verify whether these relationships were mediated by an external variable. A moderation analysis was also done to estimate if the effect of such a variable could influence the strength and direction of these relationships after verifying that the required assumptions were met.

## 3. Results

Figure 1 depicts the flow chart of patient recruitment, as well as different outcomes.

The median age of the whole sample was 74 years and 54% were men. The median period of hospitalization was 9 days.

During hospitalization, all patients were given a mean dose of 20 mg of steroids (methylprednisolone) once a day.

Compared to survivors, patients deceased or admitted to ICU showed higher median levels of NLR, CRP, leukocyte, and neutrophil count, and lower values of lymphocyte count and P/F ratio. Regarding age, significant differences were only observed between deceased and survivors.

As regards comorbidities, no statistically differences were observed between the various outcomes. All demographic and clinical characteristics of the patients are shown in Table 2.

Outcomes were statistically different (*p* < 0.01) from baseline at median time and at the end of hospitalization.

**Figure 2:**
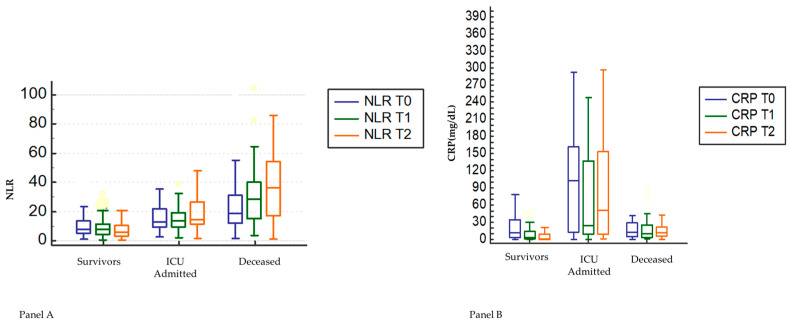
Time course of median values of NLR (Panel (**A**)) and CRP (Panel (**B**)) in survivors, deceased and ICU-admitted patients during hospitalization. NLR: neutrophil-to-lymphocyte ratio. Differences between subgroups (survivors, ICU-admitted, deceased) are statistically significant (*p* < 0.005). Differences between median values of NLR in deceased and median values of CRP in ICU-admitted are statistically significant (*p* < 0.05) during time course (T0, T1, T2).

First, we analyzed the temporal trend shown by NLR and CRP during hospitalization and assessed the differences by repeated ANOVA measures, in the whole sample and for each subgroup (Figure 2A,B).

While the whole sample, survivors, and ICU-admitted patients showed a flat pattern of NLR over time, deceased patients, by contrast, showed an increase of NLR from baseline over time. At variance, CRP tended to fall from baseline to median day of hospitalization in all four subgroups, but steeply increased at the end of hospitalization only in ICU-admitted patients.

As shown in Figure 2, admission to ICU occurred within an interval corresponding to the peak of CRP, while death occurred within the interval in which the maximum peak of NLR was observed.

**Figure 3:**
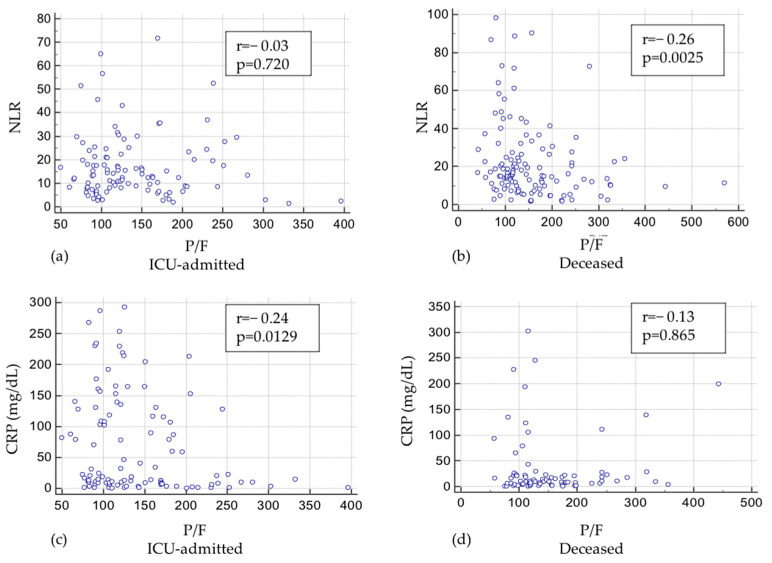
Pattern of relationships between NLR and CRP and P/F with different outcomes (Panel (**a**): NLR and P/F in ICU-admitted; Panel (**b**): NLR and P/F in deceased; Panel (**c**): CRP and P/F in ICU-admitted; Panel (**d**): CRP and P/F in deceased).

The correlation between NLR and P/F was not statistically significant in ICU-admitted patients (r = −0.03, *p* = 0.7203, Figure 3a) but was significant in deceased patients (r = −0.26, *p* = 0.0025; Figure 3b). An opposite phenomenon was observed in the correlation between CRP and P/F ratio in the two subgroups identifying the outcome (r = −0.24, *p* = 0.0129; r = −0.13, *p* = 0.865) (Figure 3c,d).

Furthermore, we analyzed these relationships using multiple linear regression analysis, with the P/F ratio as the dependent variable, and then used univariate and multivariate logistic regression analyses with single outcomes as dependent variables (Table 3 and Table 4). All multivariate regression models were adjusted for age, sex, and comorbidities.

In the multiple linear regression model (Table 2) adjusted for age, sex, and comorbidities, NLR and CRP significantly predicted P/F in both whole population and in survivors, while only CRP predicted P/F in ICU-admitted patients; conversely, only NLR predicted P/F in deceased patients.

In Cox proportional hazard regression, NLR predicted mortality independently of CRP, which was not statistically significant, as well as other confounders. ICU admission was significantly associated with both biomarkers, although CRP showed a higher HR, a more significant *p* value, and a more restricted confidence interval (Table 4).

Based on our results, showing that NLR and CRP are differently associated with P/F in deceased and ICU-admitted patients, we wondered whether the relationship between CRP and P/F could be mediated by another variable that could partly explain why CRP has a greater weight than NLR in influencing P/F in ICU patients, with no significant association with P/F in deceased patients.

**Figure 4:**
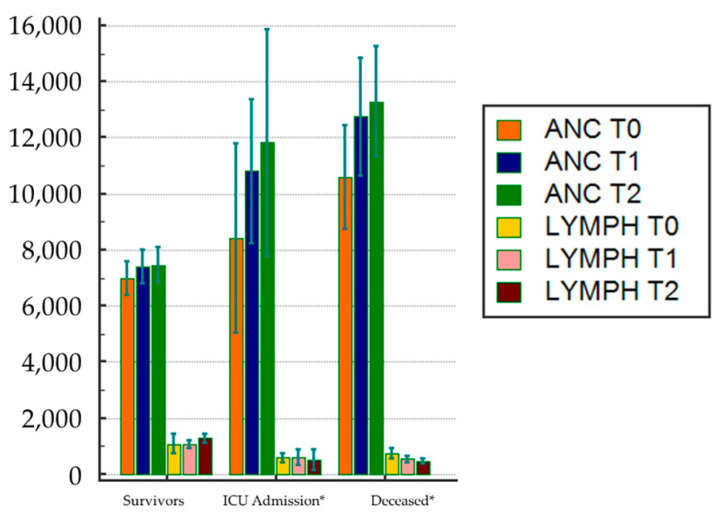
Repeated ANOVA measures clustered by outcome groups. Bars represent 95% confidence intervals (CI). ANC: absolute neutrophil count; T0: baseline; T1: median day; T2: last day of hospitalization. * Significant variation between groups (*p* < 0.001).

Observing the trend of NLR over time, we used as mediating variables the two cell populations from which it is calculated, namely absolute neutrophil and lymphocyte counts. We first demonstrated, by repeated ANOVA measures, that the mean levels of neutrophils and lymphocytes differed significantly at the various time intervals, when comparing patients grouped for outcome (Figure 4).

**Figure 5:**
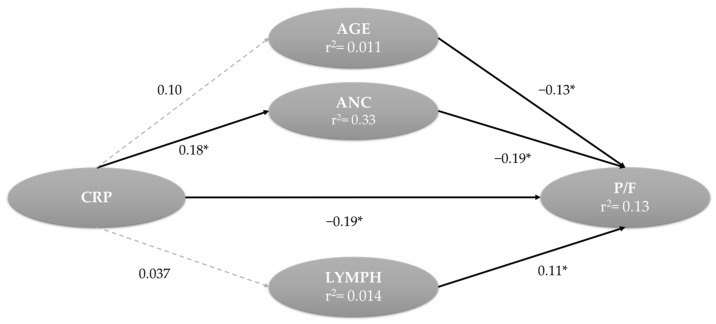
Path analysis performed in 764 patients with COVID-19 using a path weighting scheme. Effect of neutrophils, lymphocytes, CRP, and age on P/F ratio. Age, neutrophils, lymphocytes, and CRP were included as independent variables; neutrophils, lymphocytes, and age had a dual relationship as both dependent and moderator variables; P/F was the dependent variable. Significant (* *p* < 0.05) direct effects are reported as continuous black lines; nonsignificant direct effects are reported as dotted lines. Arrows indicate the direction of the effects tested in the model. r^2^ indicates the variance explained by the model.

Finally, mediation analysis was undertaken to examine the mediating effect of neutrophils, lymphocytes, and age on the relationship between CRP and P/F. Age, ANC, CRP, and lymphocytes significantly and directly influenced P/F, while the influence of CRP on P/F was also mediated by ANC, with no mediating effect of lymphocytes and age (Figure 5).

Table 5 shows details of the significant indirect effect of CRP on P/F, mediated by neutrophils (b = −0.035, *p* = 0.001).

Furthermore, the direct effect of CRP on P/F in the presence of the neutrophils as mediator was stronger (83.7%; b = −2.849 *p* < 0.001) when compared to the indirect effect mediated by neutrophils (16.3%; b = −0.035; *p* = 0.001). Hence, these results suggest that neutrophils partially mediated the relationship between CRP and P/F.

## 4. Discussion

To the best of our knowledge this is the first study evaluating the pathways of the main immuno-inflammatory circulating biomarkers, as well as their direction, in COVID-19 patients. Notably, we demonstrated the relationships between two markers that we have already described as prognostic factors in COVID-19 patients [14], namely NLR and CRP, with P/F, which is in turn a marker of respiratory failure secondary to respiratory distress.

Recently, Sinatti et al. [15] highlighted the potential of PaO_2_/FiO_2_ in predicting pneumonia progression towards ARDS in COVID-19 patients.

Notably, the acute respiratory failure in COVID-19 is characterized by a specific pathological substrate: the primum movens is endothelial damage, as a consequence of the hyperinflammatory state, with a massive cytokine and immune cell storm. In this sense, ARDS is the first result of a V/Q mismatch due to a vascular damage [16]. On this topic, recent studies have highlighted the link between inflammation, arterial stiffening, and cardiovascular events [17]. Furthermore, our group [18] recently demonstrated the stiffening of elastic arteries following COVID-19 infection, followed by partial regression in survivors, in contrast to a higher brachial-ankle pulse wave velocity (PWV) shown in deceased.

Overall, the mechanisms underlying the close connection between respiratory failure and systemic inflammation are still under debate. We aimed to investigate this link in order to identify the time course and dynamics of the release of inflammatory substances and activation of the immune system during hospitalization, which should be considered as an alarm bell.

Laboratory abnormalities have been linked to adverse outcomes in COVID-19 patients. In our previous study [14], we assessed the predictive value of inflammatory biomarkers, such as NLR and CRP, on mortality and severe COVID-19 disease by examining their trends during hospitalization. In this study, we demonstrated different CRP and NLR behavior as related to mortality and ICU admission.

In the present study we attempted to understand whether these two biomarkers are differently involved in determining outcome, and to identify the relationships, if any, between both CRP and NLR with P/F ratio. We demonstrated for the first time the influence of CRP and NLR on P/F, giving ground to the concept that a derangement in host immune-inflammatory response can influence disease severity.

CRP is a non-specific acute phase protein, induced by IL-6 in the liver, and is a sensitive biomarker of inflammation, infection, and tissue damage. The increase of CRP levels generally starts as early as 4–8 h after the initiation of the inflammatory process and peaks at 48 h, although the duration of this peak is variable and directly proportional to the stimulus’ persistence, with a half-life of about 19 h [19]. The higher CRP levels observed in COVID-19 patients requiring intensive treatment is the consequence of a systemic hyper-inflammatory state occurring in severe COVID-19 cases [20]. Despite its low specificity, CRP is a helpful marker in a lot of acute conditions characterized by inflammation/infection [12], and its elevation was shown to be associated with a bad outcome in cardiovascular diseases [21,22]. It is crucial to emphasize, in this respect, the functional role of CRP in the inflammatory process, which is aimed at recruiting complement components with positive feedback on inflammation, especially involving the endothelium [23,24,25]. In COVID-19 patients, CRP has been already described as a bad outcome predictor, together with the cytokines associated with its expression (IL-6, IL-10) [26,27].

In the present study, the prominent peak in NLR (Figure 2, Panel A) should be finalized to clear the virus. However, it is likely that the early activation of the innate immune response (increase in neutrophils) and the subsequent fall in lymphocytes (adaptive immune response) characterizing the host response to SARS-CoV-2 infection, resulted in it being unable to fulfil the purpose [28].

Lowery et al. [29] described the kinetics of the innate immune system, demonstrating that COVID-19 pathophysiology depends on the so-called cytokine storm, with production of cytokines and chemokines (TNF, IL-6, CXCL10, CCL2, CCL5 and IFN-II), and/or the lack of early IFN-I and IFN-III expression. The consequent prolonged activation of the innate immune system and a continuous suppression of lymphocytes released into circulation are main characteristics of COVID-19 disease. An elevated absolute neutrophil count (ANC) has been described as a negative predictor of outcome in COVID-19 patients [30,31], reflecting the hyper-activation of the innate immune response caused by both virus-triggered or cytokine-dependent mechanisms [13]. In particular, neutrophils are involved in platelet activation, over-production of inflammatory cytokines, and epithelial and endothelial cell damage, especially through a process named NETsosis, which is a molecular mechanism leading to the formation of neutrophil extracellular traps (NETs) [32]. An elevated expression of NETs could be associated with death and disease progression in COVID-19 patients [33].

By contrast, adaptative immune response induces a reduction of lymphocyte absolute count in COVID-19 as a consequence of extended TNF-α-induced apoptosis, peripheral consumption, direct ACE-2-cytopathic effect, or through the interaction with CD147 [29,34,35,36]. Peripheral lymphopenia in COVID-19 patients has been largely described in recent literature assessing its predictive value for disease severity and mortality [37,38]. In addition, neutrophilia itself leads to a suppression of lymphocytes through a cytotoxic indirect effect [39,40].

NLR represents the balance between innate and immune response. It readily increases as a consequence of a physiological and pathophysiological response to acute stress [29]. NLR may be considered a marker of subclinical inflammation, with higher values in acute exacerbations such as CAP, COPD, sepsis, cancer, and many cardiac diseases [41,42,43,44,45]. It is strictly linked to the immune system derangement and could be used as a predictor of disease severity and mortality, especially in conditions characterized by systemic inflammation, such as COVID-19 [14,46,47].

Considering that respiratory failure in COVID-19 is characterized by a strong inflammatory involvement, we sought to identify a potential link between the host response to viral load and severity of the degree of COVID-19 disease. Some studies have already identified an association between CRP and respiratory failure. Poggiali et al. [48] and Herold et al. [49], in two single-center studies, demonstrated that higher CRP levels are associated with lung function worsening, but data on their time course throughout hospitalization and their correlation with outcome were lacking. On the other hand, Mueller et al. [50], in a single-center study, showed the temporal relationship between CRP and P/F without any data referred to outcome.

In a recent study, we emphasized that NLR independently predicted mortality and ICU admission in COVID-19 patients, however, this prediction was abolished after adjustment for P/F [14].

Here, we further tested the pathways linking neutrophils, lymphocytes, age, and CRP to P/F. In consideration of the differences in the time course of NLR and CRP between ICU-admitted and deceased during hospitalization (Figure 2A,B), we investigated the relationships of these biomarkers to P/F, according to outcome, and found a significant inverse correlation between NLR and P/F in deceased (Figure 3b) and a significant inverse correlation between CRP and P/F in the subgroup of ICU-admitted (Figure 3c).

Furthermore, we corroborated the robustness of these correlations with a multiple linear regression analysis, demonstrating that NLR and CRP significantly predicted P/F in the whole population, while only CRP predicted P/F in ICU-admitted patients, and only NLR predicted P/F in deceased patients (Table 2). These findings could imply that inflammation plays a key role in the worsening of respiratory failure, but also that a dysfunction of immune system, as shown by neutrophilia/lymphopenia, could be closely associated with decease.

The clinical implications of our findings look interesting. For each increase of one unit of standard deviation of NLR, the risk of mortality increased by 77% in the whole sample. As to ICU admission, NLR and CRP both have a specific weight, especially CRP whose increment of one unit of standard deviation corresponded to a 70% increase in the risk of ICU admission.

Finally, we investigated the pathways and their influence on P/F. In this way, we demonstrated that mean levels of neutrophils and lymphocytes, whose absolute counts determine NLR, have a significantly different trend during hospitalization in ICU-admitted and deceased (Figure 4). Besutti et al. [51] have recently reported that persistent lung abnormalities had a link to inflammatory burden, especially to CRP, reflecting the intensity and the duration of the inflammatory reaction. In keeping with Besutti’s data [51], we considered the potential influence of neutrophils and lymphocytes on the relationship between CRP, a marker of inflammation, and P/F, a marker of lung function.

We observed that, on the worsening of lung function, CRP had a large direct effect (83.7%) (Table 4) on P/F: the higher was on CRP at hospital admission, the lower was P/F (Figure 5) (path coefficient: −0.19; *p* < 0.05). Neutrophils significantly mediated the deleterious effect of inflammation on lung function. In fact, the neutrophil count indirectly influenced (16.3%) the relationship between CRP and P/F (Table 5, Figure 5). Taken together, these data support the notion that neutrophilia would potentiate the impact of CRP on P/F, therefore paving the way to the need for ICU admission.

To the best of our knowledge, our study is, therefore, the first to demonstrate some of the pathways, and their directions, involved in the pathogenetic chain of respiratory failure in COVID-19 patients. Notably, we carried out this retrospective survey involving two different centers recruiting a selected cohort of hospitalized COVID-19 patients free of confounders affecting lymphocyte and neutrophil counts and/or serum CRP values.

Our study has some strengths: the ability to demonstrate in two different centers (a) the different pattern of relationships existing between NLR and CRP with P/F as related to different outcome; (b) the inverse relationship between NLR and CRP with P/F; (c) the immuno-inflammatory pathways revealing the co-participation of CRP and neutrophils in determining lower P/F values. Our study also has limitations: it is retrospective, carried out on patients with homogenous demographic and clinical characteristics. Moreover, although recently in patients with ARDS it was suggested that clinicians assess P/F taking into account PEEP values [52], since many PEEP data were missing, we have decided to calculate P/F traditionally in all patients, as the ratio between pO_2_ and FiO_2_. Furthermore, as all patients were given a fixed dose of steroids (methylprednisolone 20 mg/day), we were unable to assess the predictive role of NLR on the response to steroids, as previously observed by Soliman et al. [53]. As to the possible occurrence of pulmonary embolism as a mechanism involved in respiratory failure of our patients, we were unable to rule out this confounder because an angio-CT scan was not performed.

## 5. Conclusions

In conclusion, NLR and CRP, which are cheap and widely available tools, show different time courses during hospitalization in COVID-19 patients, with a characteristic pattern depending on outcome. An imbalance between innate and adaptive immunity (increase in NLR) associated with systemic inflammation (increase in CRP) is linked to deterioration of respiratory function, with a specific prediction of outcome: NLR predicted P/F in deceased patients, whereas CRP predicted P/F only in ICU-admitted patients. The mediation analysis confirmed that CRP, neutrophils, lymphocytes, and age are linked with P/F (Figure 5) in the same pathogenetic chain leading to respiratory failure.

Further prospective multi-center studies are needed to better understand this complex pathophysiological chain, and this model should also be applied in other pathophysiological conditions with an immuno-inflammatory involvement. In addition, more information is needed to better understand the molecular basis of inflammatory response, leading to cytokine storm and respiratory function worsening in COVID-19 patients.

## Figures and Tables

**Table 1:** Baseline flow rate and FiO_2_ of oxygen supplementation. A total of 160 patients did not require oxygen support. HFNC: High Flow Nasal Cannula; C-PAP: Continuous Positive Airway Pressure; NIV: Non-Invasive Ventilation; NIMV: Non Invasive Mechanic Ventilation.

	Low Flow Oxygen Therapy			High Flow Oxygen Therapy		NIMV			
	Nasal Cannula		Venturi Mask	Venturi Mask	HFNC	C-PAP			NIV
	1–3 L/min (FiO_2_ = 0.24–0.32)	4–6 L/min (FiO_2_ = 0.36–0.44)	4–8 L/min (FiO_2_ = 0.24–0.35)	10–12 L/min (FiO_2_ = 0.40–0.60)	FiO_2_ = 0.40–0.60	FiO_2_ ≤ 0.40	FiO_2_ = 0.41–0.50	FiO_2_ = 0.51–0.60	FiO_2_ = 0.50–1.00
Patients, n	51	46	101	67	115	29	89	54	52

**Table 2:** Main demographic and clinical features of the whole sample and subsets of patients (T0 = admission; T1 = median day of hospitalization; T2 = last day of hospitalization).

	TOTAL *n* = 764	SURVIVORS *n* = 534	ICU ADMITTED *n* = 106	DECEASED *n* = 124	*p*
**Age, Years**	74 (72–75)	71 (69–73)	71 (67–73)	85 (84–86)	<0.000001
**Male Sex, *n* (%)**	412 (54.1)	282 (68.2)	69 (16.7)	61 (14.8)	0.019
**Lymph, 10^9^/L**	800 (718–800)	900 (800–900)	581 (506–820)	600 (500–671)	<0.000001
**ANC, 10^9^/L**	6500 (6200–6800)	5900 (5600–6300)	7500 (6760–8927)	9000 (7500–9039)	<0.000001
**P/F Ratio**	206 (198–224)	258 (241–272)	171(121–133)	128 (117–146)	<0.000001
**NLR T0**	8.18 (7.7–8.9)	6.7 (6.2–7.3)	13.2 (11.1–15.8)	15.5 (13.6–18.6)	<0.000001
**NLR T1**	8.7 (7.8–9.7)	7 (6.1–7.8)	13.5 (11–16)	23 (17.8–31.3)	<0.000001
**NLR T2**	8.9 (8.6–10.5)	5.2 (4.5–5.3)	13.5 (12.4–22.4)	33 (22.6–41.7)	<0.000001
**CRP T0, mg/dL**	9.4 (8.4–10.6)	7.7 (6.2–8.7)	22 (12.8–73.8)	13 (9.3–15.5)	<0.000001
**CRP T1, mg/dL**	3.3 (2.5–4.5)	1.9 (1.4–2.4)	16.2 (8.5–25)	9.7 (6.9–11)	<0.000001
**CRP T2, mg/dL**	4.9 (3–6.6)	1.5 (1.3–2.2)	52.7 (22.4–101)	10 (8.1–16.8)	<0.000001
**Length of Stay, Days**	9 (8–10)	10 (10–11)	4 (3–5)	8 (7–9)	<0.000001
**Comorbidities**					
**Hypertension n (%)**	457 (62.5)	306 (57)	68 (64)	83 (66)	0.09
**Diabetes, n (%)**	334 (43.9)	221 (41)	47 (44)	66 (53)	0.06
**CKD, n (%)**	166 (21.7)	105 (19)	30 (28)	31 (25)	0.082
**COPD, n (%)**	107 (13.8)	82 (15)	11 (10)	14 (11)	0.252
**CV Disease, n (%)**	297 (25.7)	198 (37)	47 (44)	52 (41)	0.279

**Table 3:** Multiple linear regression adjusted for age, sex, and comorbidities according to outcome.

Dependent Variable P/F Ratio	WHOLE POPULATION			SURVIVORS			ICU ADMISSION			DECEASED		
	β	r	*p*	β	r	*p*	β	r	*p*	β	r	*p*
NLR	−1.91	−0.22	<0.0001	−1.68	−0.13	0.002	−0.35	−0.007	0.438	−0.84	−2.2	0.023
CRP	−0.5	−0.27	<0.0001	−0.65	−0.24	<0.0001	−0.19	−0.23	0.015	−0.15	−1.5	0.134

**Table 4:** Univariable and multivariable Cox regression models adjusted for sex, age, and comorbidities.

DEPENDENT VARIABLE: DECEASE				
	HR Univariable	*p*	HR Multivariable	*p*
NLR	1.05 (2.01 *) [1.0406–1.0709]	<0.0001	1.04 (1.77 *) [1.0295–1.0618]	<0.0001
CRP	1.002 [0.9996–1.0058]	0.0879	1.002 (1.001 *) [0.9994–1.0063]	0.1081
**DEPENDENT VARIABLE: ICU ADMISSION**				
	**HR Univariable**	***p***	**HR Multivariable**	***p***
NLR	1.02 (1.4 *) [1.0127–1.0390]	0.0001	1.02 (1.39 *) [1.0117–1.0419]	0.002
CRP	2.66 [2.4315–3.1368]	<0.0001	2.4 (1.7 *) [1.922–2.615]	<0.0001

* HR obtained using NLR and CRP z-scores.

**Table 5:** Joint assessment of the direct effect of CRP on P/F and the mediation of neutrophils.

			95% Confidence Interval				
Effect	Estimate	SE *	Lower	Upper	Z **	*p*	% Mediation
Indirect	−0.035	0.174	−0.940	−0.251	−3.19	0.001	16.3
Direct	−2.849	0.640	−4.153	−1.583	−4.45	<0.001	83.7
Total	−3.404	0.672	−4.678	−2.112	−5.07	<0.001	100.0

* SE = standard error; ** Z = Z-score.

## Data Availability

The data presented in this study are available on request from the corresponding author.

## Abbreviations

ANC	absolute neutrophil count
C-PAP	continuous-positive air pressure
CRP	C-reactive protein
HFCN	high flow nasal cannula
ICU	Intensive Care Unit
LYMPH	lymphocytes
NIMV	non-invasive mechanic ventilation
NIV	non-invasive ventilation
NLR	neutrophil-to-lymphocyte ratio
P/F	PaO_2_/FiO_2_ ratio
CAP	Community acquired pneumonia
CKD	chronic kidney disease
COPD	chronic obstructive pulmonary disease
CV disease	cardio-vascular disease
V/Q	ventilation/perfusion
